# Recent advancements in molecular marker-assisted selection and applications in plant breeding programmes

**DOI:** 10.1186/s43141-021-00231-1

**Published:** 2021-08-27

**Authors:** Nazarul Hasan, Sana Choudhary, Neha Naaz, Nidhi Sharma, Rafiul Amin Laskar

**Affiliations:** 1 Cytogenetic and Plant Breeding Lab, Department of Botany, Aligarh Muslim University, Aligarh, U.P 202002 India; 2 Department of Botany, Bahona College, Jorhat, Assam 785 101 India

**Keywords:** Gene, DNA marker, Crop plant, TILLING; CRISPR Cas9

## Abstract

**Background:**

DNA markers improved the productivity and accuracy of classical plant breeding by means of marker-assisted selection (MAS). The enormous number of quantitative trait loci (QTLs) mapping read for different plant species have given a plenitude of molecular marker-gene associations.

**Main body of the abstract:**

In this review, we have discussed the positive aspects of molecular marker-assisted selection and its precise applications in plant breeding programmes. Molecular marker-assisted selection has considerably shortened the time for new crop varieties to be brought to the market. To explore the information about DNA markers, many reviews have been published in the last few decades; all these reviews were intended by plant breeders to obtain information on molecular genetics. In this review, we intended to be a synopsis of recent developments of DNA markers and their application in plant breeding programmes and devoted to early breeders with little or no knowledge about the DNA markers. The progress made in molecular plant breeding, plant genetics, genomics selection, and editing of genome contributed to the comprehensive understanding of DNA markers and provides several proofs on the genetic diversity available in crop plants and greatly complemented plant breeding devices.

**Short conclusion:**

MAS has revolutionized the process of plant breeding with acceleration and accuracy, which is continuously empowering plant breeders around the world.

## Background

The advent of the Green Revolution in the 1960s brought a step change in the potential yield of wheat and rice and is credited with avoiding severe food crises [1]. Since this time, there has been a constant expectation that plant breeding efforts will be able to sustain gains in yield, ironically against a background of decreasing funding (American Society of Agronomy, 2018). At the same time, new intensive cropping systems promoted by the Green Revolution resulted in increased pressure from pests and disease while farm areas continue to push into more marginal land [2]. To meet these challenges, breeding and geneticists have been very successful in identifying sources of novel genetics from pre-Green Revolution landraces with the intention of bringing various biotic and abiotic stress tolerance into high-yielding semi-dwarf backgrounds prevalent in farmers’ fields [3, 4]. The concept of MAS has been used extensively as justification to identify and clone hundreds of genes across many species [5–7]. Rice in particular boasts dozens of cloned genes with significant phenotypic effects and serves as a useful case study to understand both the potential value of marker-assisted selection and its barriers to deployment. The rich genetic variations amenable to MAS in rice and other crops is a function of the partitioning of rice genetic diversity [8, 9] and its adoption as the first model species in monocots and subsequent worldwide efforts to publish its genome sequence (IRGSP 2005). For example, the Q-TARO database currently contains 114 cloned genes with natural variants affecting various traits [10], and the number of identified QTLs is many times this value. Marker-assisted selection is a newly emerging approach due to which various problems of conventional breeding avoid and enhance the selection criteria of phenotypes with the selection of genes, either indirectly or directly. Molecular or DNA markers are not regulated through the environment and conditions have no effects in which the crop plants are grown and observable in the stages of growth of the plant. With the accessibility of a variety of molecular markers and hereditary maps, MAS has gotten conceivable both for characteristics administered by significant quality just as for quantitative trait loci (QTLs). The handiness of a given molecular marker is reliant on its ability in revealing polymorphisms in the nucleotide sequences permitting segregation between various molecular marker alleles. These polymorphisms are revealed by molecular techniques such as restriction fragment length polymorphism (RFLP), amplified fragment length polymorphism (AFLP), microsatellite or simple sequence length polymorphism (SSR), random amplified polymorphic sequences (RAPD), cleavable amplified polymorphic sequences (CAPS), single-strand conformation polymorphisms (SSCP), single nucleotide polymorphisms (SNPs) and others (Khlestkina,2014).

A fruitful utilization of molecular markers to help breeding systems depends on a few elements:

1. A molecular marker in association with a genetic map linked to a gene or quantitative trait loci (QTLs) of agronomic interest;
2. Molecular markers are tightly associated with the QTLs or major genes;
3. Sufficient recombination between the molecular markers that are associated with desirable traits and the rest of the genome; and
4. A possible analysis method of a large number of individuals in a time and cost-effective manner.

**Fig. 1:**
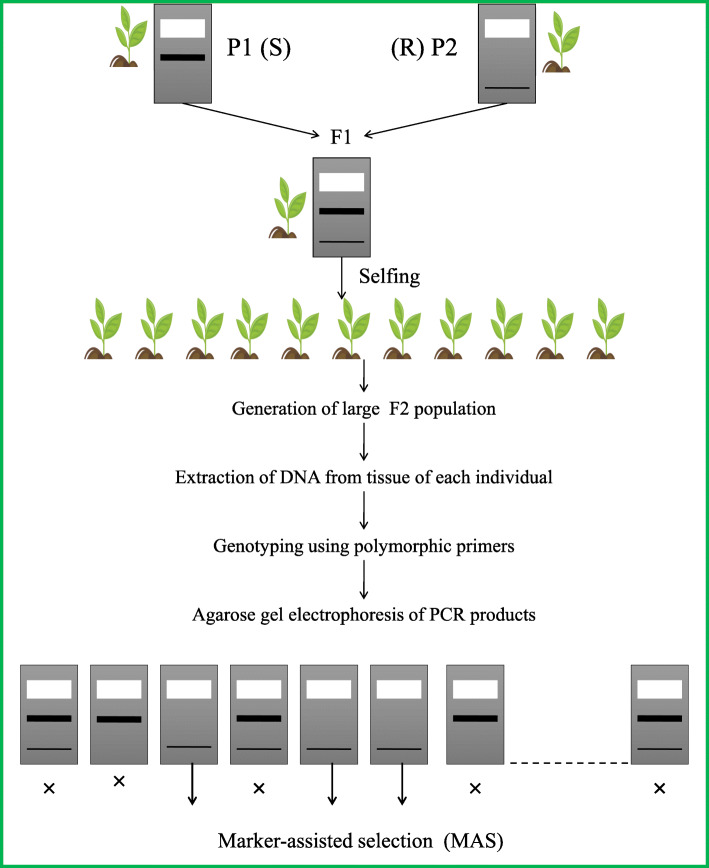
The figure explains the basic procedure of marker-assisted selection

Most of the fruitful utilization of MAS examined beneath depends on this class of molecular markers. The gene located on a particular region of the chromosome can be shown by one or more QTLs. In this situation, genomic regions to be chosen are frequently chromosome fragments; it is in this manner best either to have two polymorphic markers flanking the targeted QTL or potentially at least one marker inside the QTL genomic region. A few models identified with the two methodologies for the introgression of QTLs into various genetic systems are discussed below. Until now, MAS has been demonstrated to be powerful for generally basic characteristics that are controlled by few genes, and a few examples are given in this review on the benefits and on the effective use of MAS for this class of traits. For progressively complex traits, it has been proven that MAS is not to be more effective; a few reasons in the premise of these challenges, a few instances of fruitful utilization of MAS for quantitative characteristics, and points of view for expanding the effectiveness of MAS for QTLs are talked about. The basic procedure of molecular marker-assisted selection is presented in Fig. 1.

## Main text

### Types of DNA markers used in MAS

There are five fundamental contemplations for the utilization of DNA markers in MAS: reliability; quality and quantity of required DNA; amount and nature of DNA required; specialized methodology for marker examination; the level of polymorphism; and cost [11, 12].

#### Reliability

Markers ought to be firmly connected to the target loci, ideally under a 5-cm distance of genes. The utilization of flanking markers or intragenic markers will significantly build the reliability of the markers to foresee the phenotype.

#### DNA quantity and quality

Some marker strategies require enormous sums and a high calibre of DNA, which may some of the time be hard to get by and by, and this adds to the expense of the system.

#### Specialized system

The degree of effortlessness and the time required for the procedure are basic contemplations. High-throughput, straightforward, and snappy techniques are exceptionally attractive.

#### Level of polymorphism

Ideally, the marker ought to be profoundly polymorphic in the breeding material (for example it should separate between various genotypes), particularly in the central breeding material.

#### Affordability

The cost of DNA markers should be affordable. This infers the test ought to be modest, easy to understand, and simple to apply for effective screening of enormous samples/populations. The cost-effectiveness of the molecular marker assay should be considered to ensure the plausibility of the molecular marker-assisted selection. Sequence tagged site (STS), Simple sequence repeats (SSRs), single nucleotide polymorphism (SNP), or sequence characterized amplified region (SCAR) markers that are gotten from an explicit DNA sequence of markers (for example limitation piece length polymorphisms: RFLPs) that are connected to a quality or quantitative attribute locus (QTL) are likewise amazingly helpful for MAS [2015].

### Classification of molecular markers

DNA or molecular markers are classified in different groups on the basis of:

1. Nature of gene action (dominant or co-dominant markers);
2. Detection methods of molecular markers (PCR based molecular markers or hybridbased molecular markers);
3. Transmission mode of molecular markers (maternal organelle inheritance, paternal organelle inheritance, maternal nuclear inheritance, or biparental nuclear inheritance).

DNA or molecular markers have been developed in various kinds and all these have applied successfully in breeding and genetic activities in different agricultural crops throughout the world. In this review, brief information has provided that is related to molecular markers based on the detection methods of molecular markers. Comparisons of the important characteristics of the most used molecular markers are given in Table 1.

**Table 1:** Comparison of most widely used DNA marker system in plants

Feature and description	RFLP	RAPD	AFLP	SSR	SNP
**Genomic abundance**	High	High	High	Moderate to high	Very high
**Genomic coverage**	Low copy coding region	Whole genome	Whole genome	Whole genome	Whole genome
**Expression/inheritance**	Co-dominant	Dominant	Dominant/co-dominant	Co-dominant	Co-dominant
**Number of loci**	Small (< 1000)	Small (< 1000)	Moderate (1000s)	High (1000s–10,000s)	Very high (> 100,000)
**Level of polymorphism**	Moderate	High	High	High	High
**Type of polymorphism**	Single base change, indel	Single base change, indel	Single base change, indel	Changes in length repeat	Single base change, indel
**Cloning and/or sequencing**	Yes	No	No	Yes	Yes
**Type of probes/primers**	Low-copy DNA or cDNA clones	10 bs random nucleotides	Specific sequence	Specific sequence	Allele-specific PCR primer
**PCR-based**	Usually no	Yes	Yes	Yes	Yes
**Radioactive detection**	Usually yes	No	Yes or no	Usually no	No
**Reproducibility/reliability**	High	Low	High	High	High
**Amount of DNA required**	Large (5–50 μg)	Small (0.01–0.1 μg)	Moderate (0.5–1.0 μg)	Small (0.05–0.12 μg)	Small (> 0.05 μg)
**Genotyping throughput**	Low	Low	High	High	High
**Cost**	Moderate to high	Low	Moderate	Moderate to high	High
**Marker index**	Low	Moderate	Moderate	Moderate to high	Moderate
**Time demanding**	High	Low	Moderate	Low	Low
**Number of polymorphic per loci**	1.0–3.0	1.5–5.0	20.100	1.0–3.0	1.0
**Primary application**	Genetic	Diversity	Diversity and genetic	All purposes	All purposes

### DNA markers

Molecular markers are sequences of nucleotides and can be explored through the polymorphisms present between the nucleotide sequences of various people. Deletions, insertions, gene mutation, duplication, and translocation of these nucleotide sequences are the basis of polymorphisms among the population; however, they do not really influence the function of genes. A perfect DNA marker ought to be co-predominant, uniformly distributed, genome, more and having the capacity to recognize a more significant level of polymorphism.

#### Hybridization-based

DNA markers, conventional, or first-generation RFLPs, require the utilization of a properly labelled DNA probe for the selection of the specific genes of interest from the digestion of DNA samples and then, by hybridization. RFLP was the primary molecular marker strategy and the main marker framework dependent on hybridization. People of the same species show polymorphisms because of insertions/deletions (known as InDels), gene mutations, duplications, translocations, and inversions. The isolation of pure DNA from the target is the primary step in the RFLP strategy. This DNA is blended in through the cutting enzymes (restriction endonucleases) which are isolated from the target such as bacteria, human cells, etc. and a specific function of restriction enzymes identify specific nucleotide sequences along the DNA strand, and therefore they cut DNA at specific loci (acknowledgment destinations). These outcomes are an immense number of segments with various lengths.

#### PCR-based DNA markers

**Fig. 2:**
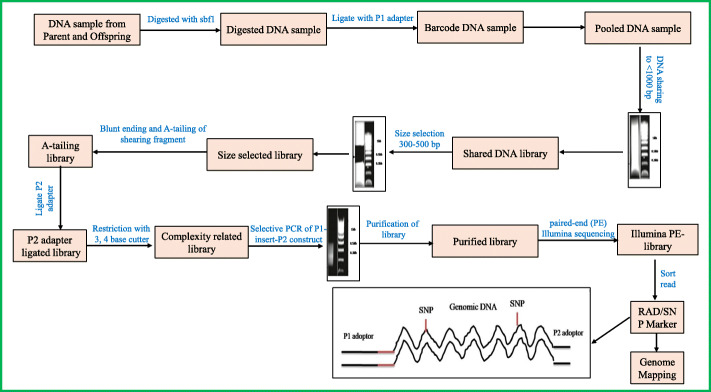
RAD-sequence: discovery and genotyping of SNPs by next-generation sequencing for genome mapping.EcoRI-MspI ligation as “adapter_P1-EcoRI” and “adapter_P2-MspI”

Molecular markers based on PCR techniques do not require a probe hybridization step. PCR-based markers are atomic markers that do not require the hybridization step. Their improvement has prompted the disclosure of a few valuable and simple to screen new generation markers, for example random amplified polymorphic DNA(RAPD), amplified fragment length polymorphism(AFLP), microsatellite or SSRs, SNP, RAMP, SRAP, ISSR, SCAR, expressed sequence tagged (EST), and so forth [13]. Being PCR-based, these molecular markers require the utilization of primer pairs for the selection of a specific part of the DNA to measure the variation in genetic material [14]. Short sequences of nucleotides that are attached with the DNA to synthesize the full length of dsDNA are called primers. Most of the primers are used for the selection of specific regions of DNA to be amplified by polymerase chain reaction and sequence analysis techniques [15]. They, therefore, start the amplification of the specific segment of DNA. After the amplification of DNA from various genotypes, the fragments of digested DNA are separated on the gel to examine the variation in the pattern of bands and further DNA fragments may be subjected to the sequencing technique for observing the sequence variation in DNA resulting in species variation [16]. Analysis of molecular markers based on PCR involves the extraction of DNA from the source and evaluation of the quality and quantity of isolated DNA and gel electrophoresis. If sequencing is required, the amplification products are usually purified, subjected to sequencing PCR, and further purified before sequencing [17, 18]. SNP genotyping through next-generation sequencing is shown in Fig. 2 [19, 20].

#### Transposable element markers

Sometimes, DNA sequences change their position in the genome and may insert into the coding regions of the genome. Such mobile DNA sequences are called transposable elements (TE). These transposable elements have been found in maize during the study of the genome by Barbara McClintock in 1950 and considered that they are present in the eukaryotic genomes at a larger scale (Bourgeois and Boissinot [21]). Before talking about the transposable elements’ markers in detail, some broad focus with respect to characterization and genomic association must be tended to. This is of extensive significance given that these strategies use the specific highlights of various TEs and differ in the properties and primer annealing sites utilized inside the transposable region. Considering their properties, TEs have been divided into class I (retrotransposons), commonly called copy and paste elements, and class II (transposable DNA), or also called “cut and paste” transposable elements [22]. The transposable elements of class I propagate with the help of the intermediate RNA molecules and form a new site in the genome, while class II transposable elements do not require an intermediate RNA molecule and excise themselves from any site of the donor and move to a specific location of the acceptor site within the genome. Since the revelation of numerous eukaryotic TEs, for example miniature inverted repeat transposable elements (MITEs), this arrangement has been tested, as it is difficult to put the new transposable elements in the current system [23].

#### Retrotransposons microsatellite amplification polymorphism

Retrotransposon microsatellite amplification polymorphism (REMAP) is also a more important marker based on retrotransposons and commonly used to evaluate the genetic diversity of individuals of the population. The protocol for utilization of REMAP is just like IRAP, although SSRs (microsatellites) are used in conjunction with specified markers of LTE at the time of PCR cycling [24]. The primers used for microsatellite loci in REMAP PCR are containing a repeated motif anchored nucleotide at the 3′ end site aiming to avoid the slippage of the primer between individual SSR motif [25].

#### Retrotransposon-based insertion polymorphism

Retrotransposon-based insertion polymorphism (RBIP) technique is used to investigate the presence or absence of sequences of retrotransposons present in the genome. In this technique, amplification of DNA is accomplished with the help of a primer having 3′ and 5′ end regions that are flanking the retrotransposon insertion site. Insertion sequences in retrotransposons are identified through the development of a primer from the LTR region. The information of nucleotide sequences along the flanking region of the retrotransposon insertion site is required in the RBIP technique, and the result of this technique in typing of a single locus compared to other molecular markers based on retrotransposons [26]. Like other molecular markers, agarose gel electrophoresis is needed for the detection of fragment polymorphism (Agarwal et al. [27]). Tagged microarray markers, which are based on fluorescent microarray scoring, were mostly utilized for high-throughput retrotransposon-based insertion polymorphism (RBIP) analysis [28].

#### Inter-SINE amplified polymorphism

Inter-SINE amplified polymorphism is based on retrotransposons having none of the LTR region and the technique has developed [29], designed specifically for potato plant. It was identified from the recent study by using bioinformatics tools that almost 6200-6500 copies of such elements founded in Solanaceae-specific short interspersed element (SINE) families and subfamilies [29]. Inter-SINE amplified polymorphism or ISAP markers are mostly based on genomic sequence amplification between adjacent SINE elements. In this technique, the primer annealed to a site other than the SINE elements and either inwardly or outwardly. For the use of the primer, different Solanaceae SINE elements compare to each other before designing the primer to ISAP. These elements are readily transferable within the genus and species of plants of Solanaceae family because they are present at a larger scale. While this technique still has not tested. ISAP described as more reproducible and useful for genotyping of different varieties of potato plants. ISAP marker system has proved that it is highly specific and while generally it is not popular in plant genetics, it represents a good attempt to utilize available genomic resources and databases. However, the design of ISAP primers requires extensive prior genomic information about SINE elements.

#### Inter-primer binding site amplification

There are limitation factors are LTR sequences must be known during utilizing retrotransposons as molecular markers. The technique inter-primer binding site (iPBS) was created by Kalendar et al. [30] to overcome the difficulties in using the PBS site in retrotransposons that have similarity with LTR transposons with a few nucleotide sequences complementarily limited to a set of tRNA [31]. Different primers with variation (12-18) in length are mostly designed to anneal the region of PBS. The retrotransposons have reverse directionality with close complexity to each other for iPBS to amplify the intergenic regions of the genome. The technique that includes the LTR region of the amplicon is a very effective method for the isolation of retrotransposons and in the scanning of the genome. Thus, this should be a more useful technique where many fingerprinting markers (e.g. REMAP, IRAP) are required or even where TEs diversity is the main objective of the study.

### Resistance gene-based markers

Several genes in animals and plants are involved in defence mechanisms and some specific sequences of these genes act as markers, so they are called resistance gene markers [32]. Before examining these markers, it is important to describe some of the features of the plant disease. Many plants developed a very active and passive defence system to ensure themselves against biotic and abiotic diseases. Active defence systems exhibit both innate and adaptive immune responses against biotic and abiotic pathogens. Innate immunity is more common in plants and animals and provides protection against several a variety of pathogens due to the action of R protein (resistance protein) and pathogen and pattern resistance receptors [33]. While in adaptive immunity, most of the defence responses of plants are regulated by interference RNA and which work mostly with viruses. Pathogens or microbes are associated with molecular patterns that are recognized by the pathogen or pattern resistance receptors (PPRs) and these receptors are conserved among the microorganisms having a place in a specific class [34]. Avr (avirulence) factors not conserved among pathogens are recognized by resistance proteins. Resistance protein (R proteins) induces signalling that produces the reactive oxygen species inside the cells and is responsible to activate the process of deliberated suicide of the cells (programmed cell death) resulting in hypersensitive reactions that kill the affected cells of the plant [35]. It was reported from a recent study of plant–pathogen interaction that the death of the cell does not restrict the spreading of pathogens; despite this, its movement is inhibited in nearby living tissues through an unknown mechanism [36]. Selected examples of gene–marker association for disease resistance in different crops are represented in Table 2.

**Table 2:** The gene–marker association for disease resistance in different crops

Species	Trait	Gene/QTLs	Molecular marker	Reference
**Wheat**	Leaf rust (*Puccinia recondita* f.sp. tritici)	Lr34 from T. aestivum	SSR	[37]
		Lr 35 from T. speltoides	STS and CAPS	[38]
	Stem rust (*Puccinia graminis* f. sp. tritici)	Sr31	STS	[39]
	Yellow rust (*Puccinia striiformis* f. sp. tritici)	Yr15	RAPD and SSR	[40]
**Rice**	Rice blast (*Pyricularia oryzae*)	Pi5(t)	CAPS	[41]
	Gall midge (*Orseolia oryzae*)	Gm7	SA598 SCAR	[42]
**Maize**	Sugarcane mosaic virus (SCMV)	Scm1 and Scm2	SCAR and CAPS	[43]
**Barley**	Barley yellow mosaic virus	rym4/rym5	SSR	[44, 45]
	Leaf rust (*Puccinia hordei*)	Rph7	CAPS	[46]

#### Resistance gene analogue polymorphism

Resistance gene analogue polymorphism (RGAP) employs uncut genomic DNA as a PCR template and degenerate primers for conserved regions of R genes to screen for R genes and RGAs [47]. Over a decade ago, in studies of crop species, it was shown that agarose gel electrophoresis is insufficient to detect most PCR fragment length polymorphisms in highly heterogeneous PCR product pools [47]. However, polyacrylamide gel electrophoresis (PAGE) yields up to a 130-fold increase in fragment length polymorphism separation capability. PAGE has been subsequently used for PCR band separation in most plant profiling studies. Based on the results of Leister et al., 1946, accurate PCR markers linked to R genes can be quickly obtained using R-like gene-specific primers. RGAP has been shown to be feasible in several areas of research. It has been used in several studies to create molecular markers for R genes that confer resistance to pathogens [48]. It has also been proven to be useful in biodiversity studies for characterizing R gene domains, namely NBS and LRR.

### RNA-based markers

Several biological responses of plants to biotic and abiotic factors during growth and developmental processes may regulate due to the expression of genes. Several methods have been evolved for getting an insight into these biological responses of plants and lead to the generation of markers based on polymerase chain reaction (PCR-based markers). Fingerprinting markers are based on the amplification of a subset of fragments prepared from RNA or DNA. The techniques summed up here depend on the transcription of a specific region of the genome that is most likely functional. Recently, Rustogi and Gupta [49] reviewed molecular markers obtained from the expressed/transcribed regions of genomes. These are treated here likewise on the chance that they use cDNA or ESTS. The techniques portrayed here may use the RNA pool straightforwardly, or after further preparing, utilizing cDNA or ESTs combined with bioinformatics instruments to create specifically or randomly designed primers.

#### Inter small RNA polymorphism

Endogenous noncoding small RNAs consisting of 20–24 nucleotides are ubiquitous in eukaryotic genomes, where they play important regulatory roles Gui et al. [50] and they provide an excellent source for molecular marker development. The flanking sequences of small RNAs are conserved, allowing the design of primers for use in PCR reactions and fingerprinting. The technique developed by Gui et al. [50] termed Inter small RNA polymorphism (iSNAP), exploits this feature. The basic principle is to use primer pairs of flanking small RNAs to initiate a PCR reaction and detect length polymorphisms that are due to InDels present in the small RNA pool [51]. According to the authors, the technique is reproducible, representing a high-throughput, noncoding, sequence-based marker system. It can be used for genome mapping and for genotyping.

#### EST-SSR

Sequencing of cDNA creates a lot of data, presently accessible in open information databases. Expressed sequences tags (ESTs) are short regions of transcribed sequences that are typically read in a direction and provide a precise way of gene expression analysis and assist in detecting genetic diversity. When changed over to cDNA, the expressed genes can be sequenced in two ways, delivering 5′ and 3′ ESTs. The last fall more frequently inside untranslated regions (UTRs), while 5′ ESTs are related to protein coding. Numerous accessible bioinformatics apparatuses [52] permit these databases to be searched to create EST-based molecular markers. The ongoing increment in the accessibility of expressed sequence tag (EST) data has encouraged the advancement of microsatellite or simple sequence repeat (SSR) markers in various plant species [53]; EST-SSRs do not vary from normal genomic (gSSR) microsatellites in their identification of amplification, the significant differences in the development and specific location of primers, as ESTSSRs are produced from the transcribed regions of the genome. They are collected legitimately from sequence data utilizing the in silico method. Data mining can be completed in numerous elective databases intended for a specific group of plants, for example Triticeae, or all more regularly in NCBI-EST.

### Targeted fingerprinting markers

Exploiting the genomic components, a novel group of markers has been created, here named targeted fingerprinting markers (TFMs). They are defined as multi-locus markers, produced in a semi-arbitrary and targeted way at different regions of the genome, and apparently compared to polymorphic sites of any gene or gene-related area regardless of their capacity. This implies the marker systems assembled here are (gene)-targeted markers which do not really yield fingerprints engaged with phenotypic mutations. TFM markers will in general combine the advantageous features of different techniques, highlights of a few essential procedures, while likewise the integration of several methods to identify genetic discontinuities or distinctiveness. Fingerprints are created in a semi-arbitrary way, because of the incorporation of normal features of the plant genome, banding designs are delivered to unknown yet targets sites. This empowers the entire genome distribution and preferable reproducibility can be accomplished with a specific primer design or even with a changed PCR protocol. Exploiting the basic genomic features makes TFM procedures effectively adaptable between numerous life forms and gives options in contrast to past AAD markers.

#### Promoter anchored amplified polymorphism

The promoter regions that facilitate the transcription of a gene are located too close to a particular gene [54]; along these lines, they can be utilized to be specify the profiling of the genome of the investigated organism. The promoter element of genes determines the point of transcription initiation and change and the specificity and rate of transcription [55]. The architecture of promoter sequences of a specific gene exhibits high diversity, comprising of many short motifs that act as the recognition site for proteins having great importance in transcription initiation [56]. This element of promoters makes them reasonable for labelling with degenerate primers to create length polymorphisms, effectively noticeable by electrophoresis. Ache et al. designed a few short oligonucleotide primers containing the degenerate sequences of cotton (*Gossypium* L.) promoter regions.

#### Direct amplification of length polymorphisms

This technique, developed by Desmarais et al. [57], resembles AAD but detects a larger number of polymorphisms and simplifies the procedure for recovering the resulting banding patterns. It also has the advantages of high-resolution fingerprinting in that it offers the possibility of directly sequencing each new marker locus [58]. It was designed to obtain nucleotide sequence information for DNA fragments from any genome with no a priori sequence data. For PCR amplification, the universal sequencing primer “M13–40 USP” is incorporated in the oligonucleotide set as a core. Selectivity is ensured by adding further bases to the 3′ end of the primers, which are termed “selective primers”. The reverse primer is also common “M13” which is a standard used in primer paired reactions. Primer sets with any desired length can be designed by varying the composition of 3′ bases in the selective primer.

#### Targeted region amplified polymorphism

The second technique, called targeted region amplified polymorphism (TRAP) and developed by Hu and Vick [59], is like SRAP but is based on a priori sequence information. The PCR conditions are the same as described for SRAP, with the priming and amplification procedure having the same rationale. The PCR reaction consists of a fixed and arbitrary SRAP primer incorporating the aforementioned modifications, i.e. selective nucleotides, filter sequences, and AT- or GC-motifs. The fixed primer is designed from available partial sequences of candidate genes, such as expressed sequence tags (ESTs). The generation of fixed primers limits the use of this technique to species where ESTs are known or requires the generation of new sequence information for primer development. Despite this limitation, it has been widely used for several purposes in different plant species [60]. Based on the use of ESTs to design primers, this method could also be placed in the RNA-based marker group, although it shares many common features with SRAP.

#### Start codon targeted

Some of the transcribed regions of molecular markers within the genome could have applications in genotyping of plants as they unmask the polymorphism that is related directly to the function of genes. Start codon targeted polymorphism (SCoT), a novel system of marker, gets popularity quickly after being described by Collard and Mackill [61]. The SCoT technique is depending on the observation that a short region of conserved sequences of the plant is mostly surrounded by ATG start codons of translation. A single primer is designed in the ScoT technique with annealing the flanking region of the initiation codon on both sides of the DNA strand. Amplified fragments are distributed within the gene having both minus and plus strands of DNA. The function of primers used in the SCoT technique is advocated by Gorji et al. [62]. These markers are more reproducible and the length of primers and annealing temperature are not the factors that determine the reproducibility of markers [62, 63]. Most SCoT markers are dominant, while co-dominant markers can also be developed during the amplification process, and thus, they could be used in the analysis of genetic diversity. These markers have been used either in isolation or in combination with different techniques to evaluate the diversity in genetic makeup and to understand the processes and structures of the population across different families of the plant [63].

#### Conserved region amplification polymorphism

Conserved region amplification polymorphism (CoRAP) by Wang et al. [64] is a technique based on the utilization of an arbitrary and fixed primer. Both SRAP and TRAP use the same kind of arbitrary primer, although CoRAP is much like TRAP to the utilization of a fixed primer and this primer is directly generated from the targeted ESTs. This is the only difference in arbitrary primers which have different core sequences (CACGC), mostly found in the intron regions of the plants. The intron core sequences ensure the utilization in genotyping of plants, while a fixed primer target coding sequences, in association with these generate more reproducible and very reliable fingerprinting. This is the advantage of CoRAP and TRAP that both derived from ESTs and have specific binding sites on the exon of targeted sequences; in spite of this, the arbitrary primers mostly bind to other exon regions (TRAPS) or to most of the introns at the time of PCR amplification. If these gene elements are accurately distributed to allow the successful PCR, the banding patterns obtained from fingerprinting will be amplified. Indels in these regions will certainly generate different distributions of amplified products. If two individuals are very close, the banding pattern resulting from the PCR product will be more similar.

### Applications of MAS in plant breeding

The advantages of MAS described below may have a deep impact on crop plant breeding in the future and may change the crop plant breeding paradigm [65]. In this review, we defined the use of molecular markers in plant breeding methodology and more emphasized the importance of marker-assisted selection schemes. We have classified these schemes into various broad areas: evolution and phylogeny, marker-assisted evaluation of breeding material; cultivar identity/assessment of purity; assessment of genetic diversity and parental selection; study heterosis; identification of genomic regions under selection; marker-assisted introgression; marker-assisted backcrossing; markers assisted pyramiding; early generation selection; and combined MAS, although there some similarities between all these categories. In general, for the development of a line, molecular markers have been integrated into the conventional scheme of plant breeding or used for substitution of conventional phenotypic selection.

#### Cultivar identity/assessment of “purity”

Seeds from different strains are to be often mixed because of difficulties in handling many seed samples that are utilized between and within plant breeding programmes. Markers may be utilized in conferring the actual identity of plant individuals. High-level genetic purity and their maintenance are more important in the production of cereal hybrids to exploit heterosis. In hybrid rice, SSR and STS markers were used to confirm purity, which was considerably simpler than the standard “grow-out tests” that involve growing the plant to maturity and assessing morphological and floral characteristics [66].

#### Study of heterosis

It was reported from a hybrid of maize and sorghum that molecular or DNA markers have been used to define a heterotic group that may be used in the exploitation of heterosis (hybrid vigour). The hybrid line is developed for the utilization in producing superior hybrids and this developmental process of the line is more time consuming and more expensive. Unfortunately, it is not yet possible to predict the exact level of heterosis based on DNA marker data, although there have been reports of assigning parental lines to the proper heterotic groups [67]. The ability of utilizing little subsets of DNA marker information is combined with phenotypic information to select a heterotic hybrid and hybrids has likewise been proposed [68].

#### Identification of genomic region selection

Distinguishing proof of movement in allele frequencies inside the genome can be significant data for breeders since it makes them aware of screening explicit alleles or haplotypes and can be utilized to configuration proper breeding procedures [69]. Different utilizations of the recognizable proof of genomic districts under choice are for QTL mapping: the areas under selection can be focused for QTL investigation or used to approve recently identified marker–trait affiliations [70]. At last, information on genomic loci selection can be utilized for the advancement of new assortments with specific allele blends utilizing MAS plans, for example, marker-assisted backcrossing or early generation selection [71].

#### Assessment of genetic diversity and parental selection

Plant breeding programmes are greatly depending on the high level of diversity in the genetic material in attaining progress in the selection process. Expansion of the genetic base of the core breeding material needs the identification of various strains for hybridization with elite cultivars [72]. Numerous studies investigating the assessment of genetic diversity within breeding materials for practically all crops have been reported. Molecular markers have been an essential tool for the characterization of genetic resources and they provide more detailed information to plant breeders for assisting in the selection of parents. In some cases, sequence information to a specific location (e.g. a specific resistance gene or quantitative trait loci) within the genetic material of the target is more desirable. For example, the comparison of marker haplotypes has enabled different sources of resistance to Fusarium head blight, which is a major disease of wheat worldwide, to be predicted [73].

#### Marker-assisted introgression

Introgression essentially implies the transfer of a particularly desirable trait from one plant species to another with the help of hybridization and frequent backcrossing. Hospital F. 2009 [74], characterized introgression as the procedure where an objective quality or QTL from a plant in population “A” is embedded to another plant in population “B” by intersection both and afterward more than once backcrossing to “B” which is known as the beneficiary and additionally repetitive parent. In this situation, DNA markers are valuable in controlling the nearness of the objective quality or QTL. This is likewise helpful in enhancing the recovery of the background genome to the recipient. Introgression utilizing molecular markers is exceptionally successful in joining genes or QTLs from landraces, on the grounds that the time required creating an improved assortment and the issue of linkage drag is diminished [75].

#### Evolution and phylogeny

Some time ago, the primary study about the evolution of species or characters was dependent completely on geographical conditions and morphological variation among the populations. The development of various techniques in molecular biology offers more information to the genetic makeup of an organism. Nowadays, a large number of molecular markers are required for phylogeny to get information about evolution and used to reconstruct the genetic map of an individual. Due to the simplicity and stability in genetic makeup, the molecular study of chloroplasts has extended the information about the phylogeny of an individual and make them perfect markers.

#### Marker-assisted backcross breeding

Markers can be used in backcross breeding at three phases of frontal area, recombinant, and foundation determinations. At the main phase of forefront choice, markers are utilized to select the desirable trait. The utilization of markers here is strong because a few qualities have relentless phenotypic selection methods or passive alleles whose impact could have been suppressed by the dominant genes. The recombinant selection is the second stage which includes selecting backcross offspring with the character and firmly connected flanking markers, so linkage drag can be decreased. The third stage is referred to as the selection of background and it includes selecting backcross descendants (for example offspring previously selected for the desirable traits) utilizing the background markers. In any case, the background markers must not be firmly connected to markers yet ought to be perfect rice markers [17, 76]. This infers markers can be utilized to select against the genome of the donor, for example decrease the hereditary commitment of the donor parent while quickening the enhancing the proportion of intermittent parent genome [77] (Table 3).

**Table 3:** The marker-assisted backcrossing in different crops

Species	Trait	Gene/QTLs	Foreground selection	Background selection	Reference
**Barley**	Barley yellow dwarf virus	Yd2	STS	Not performed	[78]
	Leaf rust	Rphq6	AFLP	AFLP	[79]
	Stripe rust	QTLs on 4H and 5H	Not performed	Not performed	[80]
	Yield	QTLs on 2H and 3HL	RFLP	RFLP	[81]
**Maize**	Corn borer resistance	QTLs on chromosome 7, 9 and 10	RFLP	RFLP	[82]
	Earliness and yield	QTLs on chromosome 5, 8 and 10	RFLP	RFLP	[83]
**Rice**	Early blight	Xa21	STS^a^	RFLP	[84]
	Early blight	Xa21	STS^a^	AFLP	[85]
	Early blight	Xa5, xa13 and xa21	STS, CAPS	Not performed	[86]
	Blast	Pi1	SSR	ISSR^b^	[87]
	Deep roots	QTLs on chromosome 1, 2, 7, and 9	RFLP and SSR	SSR	[88]
	Tolerance, disease, resistance, quality	Subchr9 QTL, Xa21, Bph and blast QTLs, and quality loci	SSR and STS	Not performed	[89]
**Wheat**	Powdery mildew	22 Pm genes	Phenotyping	AFLP	[90]

#### Marker-assisted pyramiding

The procedure of integrating multiple genes at the same time or quantitative trait loci into a single genotype is known as pyramiding [91]. Pyramiding of traits is extremely fundamental for broad range resistance of disease from BLB of rice and to ensure the strength against the durability of resistance. Molecular markers encourage determination because molecular or DNA-based markers are non-destructive and encoding of markers for various desirable genes can be tried utilizing a single DNA test without phenotyping. Joining numerous genes of disease resistance or QTLs to provide durability against disease resistance has been the most across-the-board utilization of pyramiding in plant breeding [92, 93]. Although it is yet conceivable to utilize traditional breeding, it is troublesome or unthinkable at an early generation because of the need to phenotypically screen each plant for all characteristics being tested. This makes it extremely difficult to assess plants from certain populations like F2 or for quality with ruinous bioassays [94] (Table 4).

**Table 4:** The gene or QTL pyramiding in different crops

Species	Traits	Gene from parent 1	Gene from parent 2	Selection stage	Marker	Reference
**Barley**	Yellow mosaic virus	rym1	rym5	F2	RFLP, CAPS	[95]
	Yellow mosaic virus	rym4, rym9	rym4, rym9	F1-derived doubled haploid	RAPD, SSR	[96]
	Stripe rust	Rspx	QTL 5	F1-derived doubled haploid	SSR	[97]
**Rice**	Bacterial blight	xa5, xa13	xa4, xa21	F2	RFLP, STS	[98]
	Bacterial blight	xa21, Bt	RC7 gene, Bt	F2	STS	[99]
	Blast	Pil, Piz-5	Pil, Pita	F2	RFLP, STS	[100]
	Brown hopper plant	Bph1	Bph2	F4	STS	[101]
	Insect resistance	xa21	Bt	F2	STS	[102]
**Wheat**	Powdery mildew	Pm2	Pm4a	F2	RFLP	[103]

Common DNA markers like restriction fragment length polymorphism (RFLP), randomly amplified polymorphic DNA (RAPD), and simple sequence repeats (SSRs) have aided in the mapping and association studies that led to the uncovering of genes of interest. These DNA markers, on the other hand, are produced at random from polymorphic locations in the genome, and some can be found far away from the gene of interest, suggesting that they are not related to the phenotype. Therefore, such random DNA markers can be replaced with functional markers (also known as perfect markers) created using polymorphic regions within genes that produce phenotypic trait variation [104]. Functional markers are directly connected to the allele of the trait of interest, unlike random DNA markers [52]. As a result, functional markers are equally preferred over random DNA markers in marker-assisted breeding (MAB). Several functional markers for plant breeding have been developed and are now being utilized in breeding programmes (Table 5).

**Table 5:** Candidate genes for functional marker development

Crop	Trait/resistance	Gene (s)	Location in chromosome	Sequence FW/REV	References
Wheat	Semi-dwarf stature	Rht-B1 and Rht-D1	4B, 4D	F-TCTCCTCCCTCCCCACCCCAAC R-CCATGGCCATCTCGAGCTGC & F-CGCGCAATTATTGGCCAGAGATAG R-CCCCATGGCCATCTCGAGCTGCTA	[105]
	Grain weight	TaSus2-2B	2	F-CGCCCTGAGCCG CATCCACA R-CGCTCGCCCGC CATTTATTTCTCT	[106]
	Grain weight	TaGW2	6	F-ATGGGGAACAGAATAGGAGGGAGGA R-CGAGTATGCCTAGAATGGAAAGAC	[107]
	Photoperiod response	Phd-H1	2	F-ACGCCTCCCACTACACTG R-CACTGGTGGTAGCTGAGATT	[108]
	Vernalization	Vrn-D4	5	F-CATAATGCCAAGCCGGTGAGTAC R-ATGTCTGCCAATTAGCTAGC	[109]
	Low molecular weight glutenin	Glu-D3 and Glu-B3	1D	F-CAGCTAAACCCATGCAAGC R-CAATGGAAGTCATCACCTCAA	[110]
	Yellow pigment content	Psy1	7A	F-ACATGCCGCTACTCCTATCC R-GTAGAGTGGCCAGACAAGGT	[111]
	Lipoxygenase gene	Talox-B1	4B	F-ATGATACTGGGCGGGCTCGT R-TCAGATGGAGATGCTGTTGGG	[112]
	Powdery mildew	Pm3	1A	F-CAAGTACCAACCACAGCCAC R-CCATTGCAACCACAGGAACA	[113]
	Stem rust resistance	Sr45	1D	F-GTCCATTTTACGACGGTCCG R-CTGGTCGGTAGGGAAGCTAG	[114]
	Drought stress tolerance	DREB1	3D	F-GAATGGATCCCGGAAAGCAC R-GGGAATGAACCAAGCCACAG	[115]
Rice	Semi-dwarf	sd1	1	F-CACGCACGGGTTCTTCCAGGTG R-AGGAGAATAGGAGATGGTTTACC	[116]
	Wide-compatibility gene	S_5_^n^	6	F-CGTCTTGCTTCTTCATTCCC R-GTAGGTAAACACAGGCAGAG	[117]
	Photoperiod-thermo-sensitive genic male (PGMS and TGMS) sterility	pms3 (p/tms12-1)	12	F-GAATGCCATCTAAACACT R-ATTTTACTCTTGATGGATGGTC	[118]
	Fragrance	badh2	8	F-AGTTATGGTCTGGCTGGTGC R-TTGTGTGCTACCCACCCTTC	[119]
	Fragrance	nksbad2	4	F-ATGGCAACATGGAAGGTAGC R-CATCAGCAAGCTCCAAACAA	[120]
	Low glutenin content	Lgc1	10	F-TTCTACAATGAAGGCGATGC R-CTGGGCTTTAACGGGACT & F-ACCGTGTTATGGCAGTTT R-ATTCAAGGGCTATCGTCT	[121]
	Fe and Zn	OsNAS3, OsNRAMP1	7	F-TCCATCGCTTGCTACCTCAC R-CCCGGAGATCGATCGAGACA & F-AGCACTCCCCCATCAATCAA R-ACTACACGGGTGGCTCTTTG	[122]
	Intermediate amylose content	Wx-in	6	F-CAGCGTCGACGTAAGCCTAT R-CAGGCCCCTGAAATCCATGT	[123]
	Bacterial blight resistance	Xa3	11	F-GAATGGGTGGGGTTGGGAAG R-CCATGCACGCTTGTCGAATC	[124]
	Brown planthopper resistance	Bph14	3	F-CAATCCGAGCTTACGTGGTG R-GGTGGAGAAGGCAAGAGTCT	[125]
	Blast resistance	Pit	1	F-GTGACGGAAGTGCATGGGTA R-ACCAGGGAACCCGACAAGAA	[126]
	Submergence tolerance	SubA1	9	F-CTAGTTGGGCATACGATGGC R-ACGCTTATATGTTACGTCAAC	[127]
	Tolerance to phosphorus (P) deficiency	Pup 1	12	F-CTGGACTTGACCCCAATGTA R-TCTGATGGAGTGTTCGGAGT	[128]
	Drought stress tolerance	OsSAPK2	5	F-AAGGACATAGGGTCGGGGAA R-TGGCCAAATGTGTGGGAGTT	[129]
Maize	Plant stature	tb1	1	F-CACATGAGCCCATGCCTCTC R-AAAGCGGTAAGTCCATGGGG	[130]
	Plant height	Dwarf8	1	F-ACACTATCACCGCTCTATTG R-ACTCTTTCCCTGACTTCATT	[131]
	Oil content	DGAT1-2	6	F-TGGCTCTGCAATCAGGAGAA R-TGAAGCAGCAAACAACGAGC	[132]
	Forage quality for digestibility	Bm3	4	F-TTCAACAAGGCGTACGGGAT R-AGTGGTTCTTCATGCCCTCG	[133]
	Provitamin A	ZmcrtRB3	2	F-GTCGGTACTGGCAAGTGGAA R-TAGTACGTGGCCATGACGTG	[134]
	Sweetness	sugary1	4	F-TCCCGACTTCAGAACGGTTG R-ACAACAGAGCAACCCCAACA	[135]
	Drought tolerance	MYBE1	5	F-GGTACCCTGTCAAGGTTCGG R-AATTACTGGCCCCAGGTTCG	[136]
Barley	Photoperiod response	Phd-H1	7	F-CCTCTTCGCTATTAC GCCAG R –GCCCTTCCCAACAGTTGCG	[137]
	Vernalization requirements	VRN-H 1	5	F-TTCATCATGGATCGCCAGTA R-AAAGCTCCTGCCAACTACGA	[138]
	Powdery mildew	NBS–LRR	2	F-CGTTTTGTATGGCGTCCGAT R-TTGTCGCTGAGGTCCATCTT	[139]
	Leaf rust resistance	Rph7	3H	F-TGGAAACCACTGTACAGCCT R-CAGGCATGGGAGTGAACCTA	[140]
	Photoperiod response	Phd-H1	2H	F-GTTGAGATCGACAGTCCCCA R-GGGCTCCTATCTCCAACTCC	[137]

### Advancement in marker-assisted selection

#### Targeting induced local lesions in the genome

**Fig. 3:**
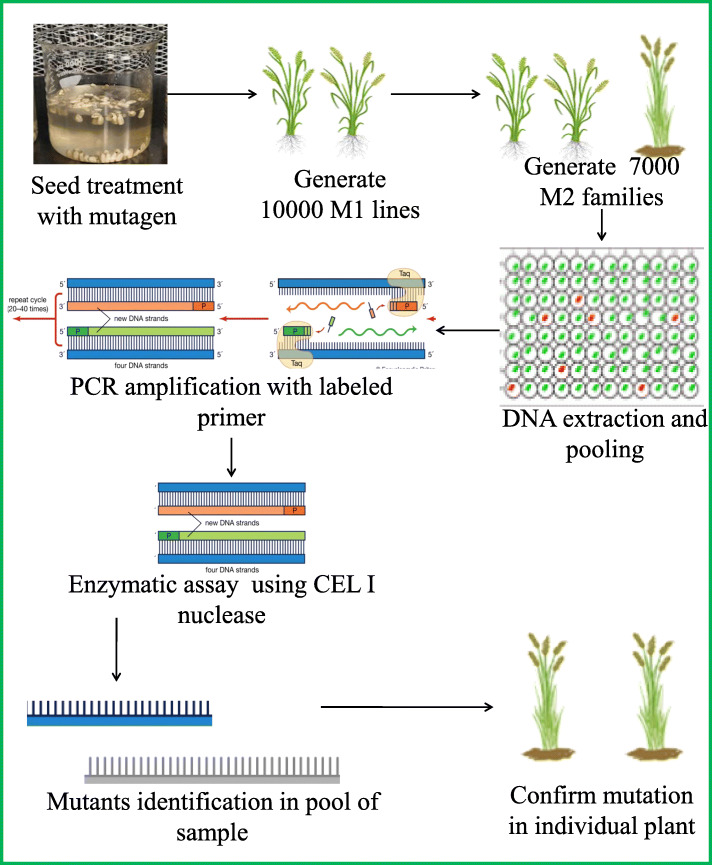
A schematic representation of traditional TILLING pathways and workflow of TILLING by sequencing

Targeted induced local lesions in the genome (TILLING) is a non-transgenic technique of reverse genetics and is applicable to most crop plants. TILLING was developed by McCallum in 1990 during the study of understanding the functioning of two genes in *Arabidopsis* plants [141]. TILLING techniques involve the first establishment of the mutagenic population through seed treatment with a standard chemical mutagen like methyl methanesulfonate (MMS) and ethyl methanesulfonate (EMS). The variations in target nucleotide sequences of mutant individuals of the population are identified through the utilization of the most important techniques such as mass spectroscopy, liquid chromatography, array-based technologies, and enzymatic mismatch cleavage [142]. Later, more important bioinformatics tools such as project aligned related sequences and evaluated SNPs (PARSESNP) are applied for the analysis of mutations induced by specific mutagens. TILLING is applicable for any of the plant species but should not be affected by the ploidy level and genome size. The most important advantage of this technique is the identification of a greater rate of gene mutations. This technique provides the precise identification of new alleles at a lower cost in a short time, so it is a time-saving bioinformatics technique that may be used in molecular genetics during plant breeding programmes. The key steps involved in TILLING are described in Fig. 3.

#### Virus-induced gene silencing

**Fig. 4:**
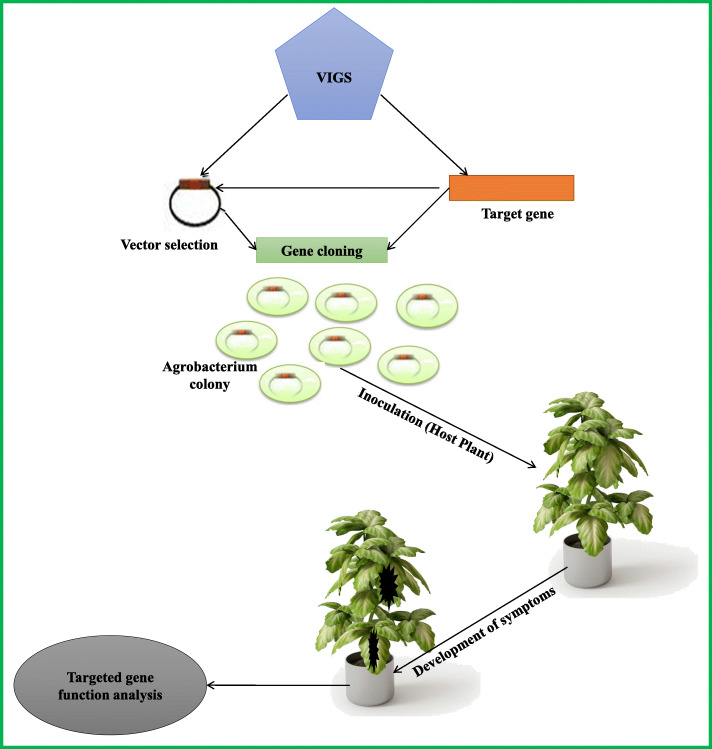
Method for high-throughput virus-induced gene silencing (VIGS). VIGS is performed by cloning a short stretch of sequence (usually 100–500 base pairs) from a candidate gene or random cDNAs into a virus genome under the control of promoter within a binary vector

Virus-induced gene silencing (VIGS) is a virus-mediated methodology that is used to exploit an RNA-mediated defence mechanism. After the synthesis of siRNA, its base pairs guide the complex of RNase in that condition in which it targets a specific single-stranded RNA that appears just like the dsRNA molecules [143]. Double-stranded RNA intermediate molecules should be processed in a way in which the siRNA present in infected cells should correspond to parts of the viral vector genome including any nonviral insert. Thus, the viral genome inserts into the cell of the infected part of the plant, then the siRNA target to the complex of RNases that corresponds to the mRNA of the host, and the symptoms of plants describe the loss of function of genes encoding the protein in infected plants [144]. The method for high-throughput virus-induced gene silencing is shown in Fig. 4. In recent years, VIGS has been applied successfully in plant reverse genomics. It is a very simple, cost-effective, and high-throughput method. Mainly, it is used in the identification of function loss of a gene of interest [143, 145]. The role of VIGS has been investigated to know the functioning of genes under abiotic stresses in different species of plants and animals. The studies about VIGS involving different model plants are not discussed in this review; despite this, the review is focused on the different crop plants. A wide variety of vectors for the VIGS technique have been developed with high silencing efficiency to expand its applications in several crop plant species for the study of genes that respond to abiotic stresses. The utility of VIGS has been demonstrated in different crop plants’ tolerance to various abiotic stresses. SlGRX1 gene silencing in tomato by a satellite DNAmβ-based VIGS vector resulted in yellowing of leaves under salinity stress compared to vector control plants due to a reduction in chlorophyll content, suggesting the role of GRX1 in salt tolerance [146]. Furthermore, the role of CaRAV1 and CaOXR1 has been studied by TRV-VIGS in chilli pepper [147]. VIGS has been used to study oxidative stress tolerance in the recent past. A few studies [147, 148] described earlier in this review that examined the role of chilli pepper genes, like CaRAV1, CaOXR1, and CaPO2, have also described oxidative stress damage in plants with these genes silenced. Silencing of CaRAV1, CaOXR1, or CaPO2 individually or co-silencing of CaRAV1/CaOXR1 in chilli pepper resulted in enhanced lipid peroxidation under stress (Fig. 4).

#### Genome editing (CRISPR)

**Fig. 5:**
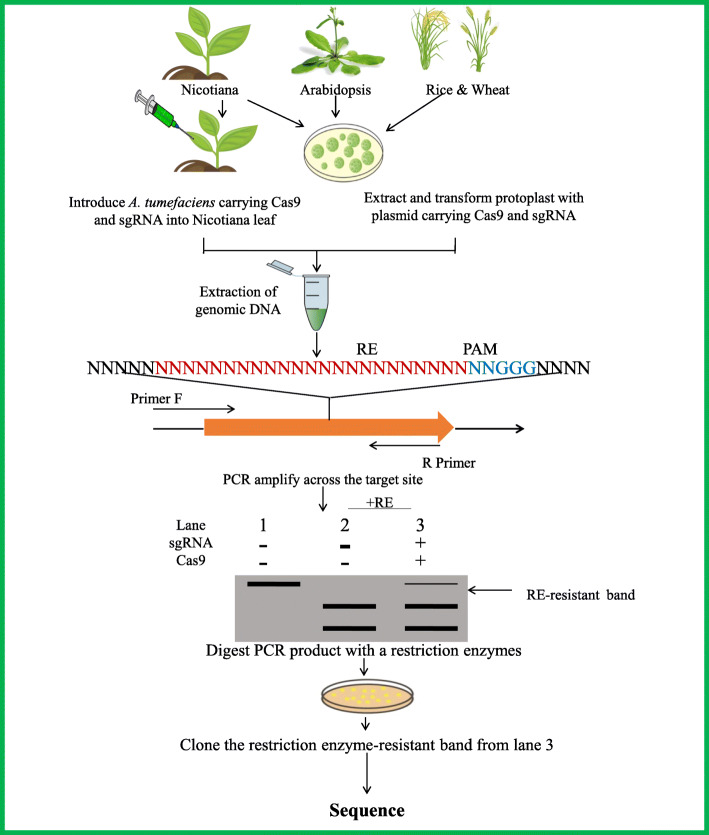
A schematic drawing illustrating an example of genome editing for crop improvement through the CRISPR/Cas9 strategy

Various crop plant has improved due to the utilization of CRISPR genome editing technique [149]. The new emerging technique of genome editing Cas9 technology is becoming the technique of choice due to its many advantages such as easy to use, ability to cleave the methylated loci, and genome editing versatility [150, 151]. CRISPR RNAs and Cas protein are the two most important parts of the CRISPR technique. Trans-encoded CRISPR RNA (tracrRNA) and CRISPR RNA (crRNA) are two short-length RNA molecules that can cleave a particular target site with the help of Cas9 endonuclease (the most explored Cas protein). Single guide RNA or sgRNA is the hybrid that results due to the artificial fusion of tracrRNA and crRNA [152]. The RNA-guided endonuclease is formed due to the joining of sgRNA and Cas proteins; this RNA-guided endonuclease mediates the cleave of a particular sequence in the genome [153]. On the basis of this Cas protein, the CRISPR–Cas system is grouped into three types: I, II, and III. Cas1 and Cas2 are two different proteins that are commonly present in all three types. Type I is present in both archaea and bacteria, while type II is only present in bacteria; however, type III is most commonly present in archaea but also in some bacteria [154]. Genome editing has been performed successfully in model plants like Nicotiana tabacum [155], Arabidopsis [156], and some economically important crops like maize [157] and wheat [158] (Fig. 5).

#### Genomic-wide association studies in crops

Genomic-wide association studies (GWAS) take full advantage of ancient recombination event to identify the genetic loci underlying traits at a relatively high resolution. GWAS methodology became well established in human genetic during a decade of great effort. With the rapid development of sequencing technologies and computational methods, GWAS are now becoming a powerful tool for detecting natural variation underlying complex traits in cops [159]. GWAS in crops usually used a permanent resource—a population of diverse (and preferably homozygous) varieties that can be re-phenotyped for many traits and only need to be genotyped once—and one can subsequently generate specific mapping population for a specific trait or QTLs in crops [160]. GWAS have now been carried out successfully in many crops, including rice, maize, foxtail millet, and sorghum [161–165]. Based on the magnitude of resources already developed and published, rice and maize are the two major models for crop GWAS, and both have panels of thousands of genotyped inbred and multiple environment trials conducted for several traits. In rice, 1083 cultivated *O. sativa* spp. *indica* and *O. sativa* spp. *japonica* varieties and 446 wild rice accessions (*Oryza rufipogon*) were collected and sequenced with low genome coverage [166]. A high-density haplotype map of the rice genome was constructed using data imputation, and a GWAS was then conducted to characterize the allele associated with 10 grain-related traits and flowering time using the comprehensive data set of 1.3 million SNPs. A GWAS was also carried out in 446 *O. rufipogon* accessions for leaf sheath colour and tiller angle, which would have stronger mapping power owing to a higher level of genetic diversity in the wild species. Moreover, the GWAS was performed based on the microarray-based genotyping approach. In total, 413 diverse accessions of *O. sativa* were genotyped at 44,100 SNP variants and phenotyped for 34 traits, and the result showed the complex genetic architecture of the traits in rice. In maize, the genetic architecture of flowering time, leaf angle, leaf size, and disease resistance traits were dissected by conducting linkage mapping and GWAS jointly in the NAM panel, and multiple related candidate genes were identified [167–169]. The GWAS result demonstrated that the genetic architecture of these traits is dominated by many QTLs with small effects. Maize oil is an important food and energy resource, and a GWAS in maize was recently performed for maize kernel oil composition [170]. A total of 368 maize lines were analysed at 1 million SNPs genome-wide, and 74 loci were found to be associated with maize kernel oil concentration and fatty acid composition. These studies show that the GWAS method in crops is a useful and robust strategy complementary to classical biparental cross mapping and has the power to genetically map multiple traits simultaneously. GWAS results are expected to be further utilized to investigate the genetic basis of plant morphology, yield, and physiology in more grasses, including close wild relatives of domesticated crops. It is important to note that GWAS have a low power for rare alleles, which make up a substantial proportion of natural variation. In rice, 44% of the SNPs are of low frequency (minor allele frequency < 0.05). In the case of rare alleles, either the use of a large sample size or the construction of multiple biparental cross population (e.g. NAM or MAGIC) may be helpful.

#### RNA-sequencing

RNA-sequencing (RNA-seq)-based genotyping retrieves genotypes from RNA-seq data. Despite the great variation in genome size for different crops, there are no significant changes in the number of genes or total gene size. Because most repetitive regions are ignored, performing transcriptome sequencing for SNP calling rather than whole-genome resequencing is quite cost efficient. For example, a recent study genotyped many SNPs and expression of QTL (eQTL) mapping simultaneously [170]. It would be much more expensive to genotype 368 maize genomes, because the repeat region occupies more than 80% of the total maize genome. RNA-seq-based sequencing has several weak points. Because the SNP density in the genic region is much lower than that in intergenic regions, the number of SNPs called from RNA-seq data may not be large enough for GWAS, especially for high-LD crops. The existence of a strong bias in SNP distribution raises another problem. In a particular tissue at a particular time point, many genes have very low or even no expression and thus cannot be used in genotyping, but RNA preparation of multiple tissues at multiple time points for a large population would greatly increase the workload. Exon sequencing-based genotyping, facilitated by exon capture, can also be applied to mapping of some large, complex crop genomes [171–173].

#### Role of MAS in crop improvement

Genetic mapping of major genes and quantitative trait loci (QTLs) for many important agricultural traits is increasing the intention of biotechnology with the conventional breeding process. Exploitation of the information derived from the map position of traits with agronomical importance and of the linked molecular markers can be achieved through marker-assisted selection (MAS) of the traits during the breeding process. However, empirical applications of this procedure have been shown that the success of MAS depends on several factors, including the genetic base of the traits, the number of individuals that can be analysed, and the genetic background in which the target gene must be transferred. MAS for simply inherited traits are gaining increasing importance in breeding programmes, allowing acceleration of the breeding process. Traits related to disease resistance to pathogens and to the quality of some crop products are offering some important examples of a possible routinary application of MAS. For more complex traits, like yield and abiotic stress tolerance, several constraints have determined severe limitations on efficient utilization of MAS in plant breeding, even if there are a few successful applications in improving quantitative traits. Recent advances in genotyping technologies together with comparative and functional genomics approaches are providing a useful tool for the selection of genotypes with superior agronomical performances.

#### MAS for yield

Although some investigations provided examples on the practical application of MAS to increase yield, it is becoming clear that an integrated approach involving traditional methods of agricultural improvement [174] and a combination of crop modelling and QTL mapping [175] are required to select crop ideotypes for a given environment. In the following selection, some selected examples of successful applications of MAS to increase yield in important crop plants like maize, rice, barley, and soybean are provided.

#### Rice

QTL alleles for yield components traits derived from the wild rice relative *Oryza rufipogon* have recently been extensively studied by using advanced BC populations [176]. In these studies, despite its inferior performance, 53% [177] and 33% [178] of the QTL allele originating from *O. rufipogon* had a beneficial effect for yield and yield components in the recipient rice elite cultivars. The lower percentage reported in the second study may be explained by a higher genetic similarity between the elite line and *O. rufipogon* at the yield QTL alleles or by the fact that in this cross the elite cultivar may have more favourable alleles at most of the identified loci. Some of the *O. rufipogon* yield QTLs identified were linked to any deleterious negative QTLs and would directly be useful to develop breeding materials. In several different instances, the *O. rufipogon* alleles showed the same effects in different genetic background and environment, supporting the stability of these yield QTLs. A thousand grain weight (TGW) QTL has recently been identified on chromosome 6 by using BC inbred line derived from a cross between the high-yield rice japonica cv. Nipponbare and the low-yield indica cv. Kasalath [179]. The QTL allele increasing TGW is derived from the low yield cv., and when introgression by MAS into a Nipppoabare NIL, this QTL increases TGW and yield per plant by 10 and 15% respectively without any effect on plant type. The genomic region in which this yield QTL is located is tagged by several molecular markers that can be used to introgress this QTL to increase yield in high-yielding rice cultivars.

#### Maize

Marker-mediated backcrossing is a selection scheme used in maize to monitor the transfer of favourable allele at QTLs (foreground selection) and to hasten the return to the recipient genotype in the remainder of the genome (background selection) (Bouchez et al., 2002). A similar approach of marker-mediated backcrossing has been used to generate series of maize NILs derived from an elite recipient line (the recurrent line) and an exotic donor line [180]. Marker-facilitated backcrossing and marker-facilitated selfing were used for foreground and background selection. As few as two BCs and one selfing (to fix the introgression segment) generations were sufficient to generate different NILs, each with different introgression genomic regions. When crossed to a tester line and evaluated in replicated field trials, different NILs revealed having received donor segments increasing or decreasing their yield performances. This breeding scheme not only creates embanked elite lines but also provides materials for the identification and mapping of yield QTLs. A possible disadvantage of this approach is that favourable epitasis effects between QTLs may not be identified. Development of a reliable method for predicting hybrid performance in maize, without generating and testing hundreds or thousands of single-cross combinations, has been the goal of numerous studies, using both marker data and a combination of marker and phenotyping data. In order to explore heterosis (hybrid vigour) and G×E interaction, Stuber et al. [180] used a cross between two widely used elite maize inbred, B73 and Mo17. They identified and mapped QTL allele that was predicted to increase hybrid yield. Markers were used to introgress the QTLs into the inbred lines, and the hybrid from the enhanced inbred lines yielded better than hybrid from inbred lines that lacked the marker-introgressed QTLs [180]. Whenever a QT for grain yield was detected, the heterozygote had a higher phenotype than the respective homozygote (with only one exception) suggesting not only overdominance (or pseudo-overdominance) but also these detected QTLs play a significant role in heterosis. This conclusion was reinforced by a high correlation between grain and the proportion of heterozygous marker. However, for the trait governed largely by additive gene action (this type of gene action might prevail for some loci affecting grain yield), the heterozygous QTL genotype would be the most favourable. For this reason, an effective prediction of hybrid performance based on markers solely would require knowledge of QTLs linked to the markers.

#### Role of MAS in stress tolerance

Besides a requirement for vernalization, overwintering crops also require frost and cold tolerance. Cold tolerance is recognized as having a complex quantitative inheritance, making therefore problematic MAS approach to increase tolerance phenotypic values. Nevertheless, few examples of successful utilization of MAS for improving cold tolerance in cold plants are available. In barley, two tightly linked QTLs for low-temperature tolerance were identified on chromosome 5H [181]; these QTLs were coincident with QTLs regulating mRNA levels as well as protein accumulation of two characterized cold-regulated (COR) genes. Several genes with the CBF transcription factor signature were mapped in a cluster in this region. Since a CRT/DRE recognition site, a potential site for interaction with a CBF transcription factor was found in the genome regulatory sequence of one of the two COR genes, the identified CBF genes represent candidates for the gene underlying the QTL [181]. Because it has been demonstrated the *CBF1* overexpression induces *COR* genes and enhances freezing tolerance in *Arabidopsis* [182], these results support the hypothesis that members of the *CBF* gene family may regulate the stress. PCR-based markers (a RAPD marker and an STS derived from the sequence of a wheat RFLP mapped in the frost tolerance QTLs region on chromosome 5H) have recently been validated for their ability in assessing frost tolerance level in two sets of winter and spring barley genotypes and in doubled-haploid lines derived from a cross between a highly tolerant and susceptible genotype [183]. These two markers were shown to discriminate efficiently between frost-tolerant and frost-susceptible genotypes. Their use in different breeding materials will clarify how much would be the gain in frost tolerance obtained only by MAS with respect to phenotypic selection in stressing environments. Rice has evolved in tropical and sub-tropical areas, and hence, its cultivation is vulnerable to low-temperature stress in temperate-growing regions and high-elevation environments. Anthers at the booting stage are known to be susceptible to low temperature, so cold stress results in delayed heading of maturation and yield reduction due to spikelet sterility. Abe et al. [184] reported the tight association of a SNP in a rice alternative oxidase gene (*OsAOX1a*) with two closely linked QTLs (*Ctb1* and *Ctb2*) for low-temperature tolerance of anther at the booting stage mapped to chromosome 4. They found that the allelic variation in molecular mass of AOX isoforms among varieties differing in low-temperature tolerance co-segregate to the presence of the QTL. These results suggest that exploitation of this SNO represent a good tool for MAS of the cold-tolerant QTLs. *Ctb1* locus has recently been physically mapped and seven candidate genes for these QTLs have been identified [185]. Aluminium toxicity is a major limiting factor for agriculture in tropical and acidic soils. Using bread wheat (*T. aestivum*) recombinant inbred lines, a single locus for Al tolerance (referred to as Alt_BH_) was found on the long arm of chromosome 4D [186]. A single gene controlling aluminium tolerance was also found in barley on chromosome 4H (Alp; Tang et al., 2000) and microsatellite markers associated with this locus have been identified (Raman et al., 2003); the microsatellite marker Bmag353 has been validated in a F3 population segregating for Al tolerance and the marker was found to predict the Al tolerance phenotype with over 95% accuracy. Previous reports showed that there is a conserved genomic region on the log arm of homologous chromosome 4 for Al tolerance among wheat (Alt_BH_), rye (Alt3), and barley (Alp) [187]. Based on common markers, it was suggested that Alt_BH_, Alt3, and Alp genes are orthologous loci because of the high level of synteny among chromosome 4DL, 4RL, and 4HL and they may share a common function.

#### Role MAS in quality traits

Most quality traits show continuous variation and influenced by environmental factors. Notwithstanding, some examples of quality traits, for which MAS is reliable approach for selection, are now available for several important crops including tomato, barley, wheat, cotton, and rice.

#### Tomato

Soluble-solids content is of paramount importance for processing tomatoes, because lines with higher sugar content require less energy input during the concentration process. To uncover the molecular basis of the sugar content variation, a QTL for total soluble solids (sugar and acids), named Brix9-2-5, derived from the green fruited tomato species *Lycopersicum pennellii*, was characterized [188]. The genetic basis of the QTL was dissected by positional cloning and was shown to be the Lin5 gene, coding for a fruit-specific apoplastic invertase hypothesized to modulate fruit sink strength. The *L. pennillii* Brix allele increases the glucose (28%) and fructose (18%) content in various genetic backgrounds of cultivated tomato and across different environments, and confer around a 3-fold increase in soluble solid content (up to 15% of the fresh fruit weight). Brix9-2-5 was shown to be partially dominant and independent of fruit weight and yield.

#### Rice

In China, there is a strong emphasis on improving the quality of indica hybrid rice varieties. “Zhenshan 97”, the female parent of several widely cultivated hybrid, is of poor quality because of its high amylase content (AC), hard gel consistency (GC), low gelatinization temperature (GT), and chalky endosperm. These three traits for cooking and eating quality are controlled by the genomic region containing the Waxy locus. The eating and cooking quality of Zhenshan 97A (male-sterile) has been improved by introgression of the Waxy gene from Mighui 63 (restorer line) [189]. MAS were used during three generations of backcrossing. An SSR marker waxy, representing the Waxy gene, was Waxy region; two RFLP markers defining a 6.1-cm interval and flanking the Waxy locus were used to select recombinant between the flanking markers and the Waxy gene (to ensure that the introgressed region was shorter than the interval defined by two RFLP markers). A total of 118 AFLP fragments were used in background selection to recover the genetic background of Zhenshan at unlinked loci. The obtained selected lines and their hybrids with Minghui 63, or Shanyou 63, showed reduced AC and an increase in GC and GT, coupled with reduced grain opacity, results from this study also confirmed that the waxy region has major effects on the three traits for cooking and eating quality.

#### Wheat

The most important quality parameters in wheat relate to the physical properties of the dough during bread making, such as extensibility and resistance to extension. These properties depend on the endosperm gluten protein, which comprises two major factors: gliadin and glutenin [190]. Generally, high molecular weight (HMW) glutenin have been found to be more important than gliadin and low molecular weight (LMW) glutenin is for dough rheological properties. Breadmaking qualities especially dough strength are dependent on the composition of HMW glutenin subunits, particularly the alleles *Glu-Alb* and *Glu-Dld.* SDS-PAGE of seed protein is used for screening wheat lines for glutenin polypeptide profiles. This method is relatively efficient because allelic variation at multiple loci can be assessed in a single gel line. PCR-based molecular markers based on sequence variation of the coding and promoter region of the wheat HMW glutenin gene at the Glu-1 locus have been developed [191]. When tested in a DH population segregating for bread-making quality, DNA and SDS-PAGE protein markers showed discrepancies of only 2 to 8.5% depending on the marker assayed. PCR-based molecular markers have also been developed for the Glu-A1 locus in Australian commercial wheat varieties [190]. These cultivars show only one or two predominant alleles at each HMW glutenin (Glu-1) homoeologous locus. The product of a single multiplexed PCR reaction permitted the discrimination of the major HMW glutenin in one simple assay. These markers are currently used in MAS for HMW glutenin in DH-based wheat breeding programmes.

#### Relation between molecular markers and function markers

The recent progress in the area of plant molecular biology and genomics has the potential to initiate a new “Green Revolution”, which is of vital importance for the development of drastically improved crop germplasm [192]. Increasingly exact linkage of markers and genes to traits will lead to more efficient plant breeding in the future [193]. Genomics technologies are being applied to the improvement of crop plants with encouraging results [72]. For over 20 years, DNA markers have been the most widely used molecular markers in crop improvement, owing to their abundance and polymorphism. Most of these markers are selectively neutral because they are usually located in non-coding and non-regulating regions of DNA [194]. The first plant DNA markers were based on restriction fragment length polymorphisms (RFLPs) [195]. The majority of molecular markers have been developed either from genomic libraries (RFLps and SSRs) or from random PCR amplification of genomic DNA (RAPDs) or both (AFLPs). However, when such markers are used for marker-assisted selection in plant breeding, they may have some limitations owing to genetic recombination giving rise to false positives [196]. Function markers (FMs) are developed from the polymorphic site within genes that casually affect the target trait variation, i.e. based on functional characterization of the polymorphisms [104]. Hence, they are more meaningful in crop improvement. It is comparatively easier to developed function markers in plants such as rice, tomato, wheat, soybean, etc. (Table 5), where either complete or nearly complete genome sequence information is available than the other in which little or no genomic information is available. The fundamental difference between functional markers (FMs), genomic molecular markers (GMMs), and random DNA markers (RDMs) is their impact on the effectiveness of the selection. FMs, GMMs, and RDMs are too limited to predict the breeding value based on limited well-associated markers. In contrast, genomic selection is based on a dense set of markers from across the genome. Meuwissen et al. [197] made a first step toward predicting a total genetic value using a genome-wide dense map of highly informative markers. GS uses the genome as the selective unit instead of using individual genetic loci that are associated with a trait. One obvious difference between GS markers and FM is the greater number of GS markers required for genotyping in a breeding population. FMs are powerful in trait-by-trait selection owing to complete linkage with trait locus alleles, which reduces the amount of linkage drag when used in combination with closely linked markers [198]. FMs allow for efficient selection of recombination between the target gene and closely linked markers in a large seedling population [198] that could significantly reduce the number of backcross (BC) generation needed. The use of RDMs also bears the risk of being lost through genetic recombination even in the presence of flanking markers. Even GMMs can be lost through the recombination [199]. Hence, the use of FMs is more efficient for gene identification and selection in breeding programmes compared to RDMs and GMMs [104].

### Advantages of MAS breeding over conventional breeding

The use of molecular or DNA markers for selection and screening of crop plants in a breeding programmes provide many advantages and therefore marker techniques are more attractive to plant breeders.

- Genotypic DNA markers can be obtained from any tissue of crop plants and investigated plants already screened at the seedling stage or even in seeds. Thus, screening and selection can be performed at an early stage for the specific traits that are expressed in the adult plants (i.e. male sterility, quality of fruit, and grain sensitivity to photoperiod). Due to the availability of information about the genotype of pre-flowering MAS allows controlling pollination, e.g. in marker-assisted recurrent selection.
- For traits with complex inheritance, every individual genetic component contributing to the trait can be selected separately. Moreover, multiple characters that would normally be epistatic (i.e. they show a certain positive or negative effect only in combination with each other) can be maintained and ultimately fixed.
- Molecular markers help in the selection of targeted alleles which are very difficult, more expensive, and/or time consuming in scoring the phenotypes (e.g. traits that are environmentally sensitive, while DNA markers are neutral to environmental changes).
- Selections can be made on a single plant basis where this would not be possible by phenotypic selection. Poor heritability does not pose a problem if the selection is based on marker information.
- Recessive genes in various crop plants can be maintained without progeny tests required in each generation, as heterozygous and homozygous crop plants can be differentiated with the assistance of codominant markers. During backcrossing, DNA or molecular markers may help minimize the linkage drag around a gene of interest and are important to reduce the generation needed to recover the genetic background of a recurrent parent.
- The time of choice of parents for crossing DNA markers can also be applied . Here, DNA markers can help in minimizing diversity in genetic makeup, and in this way, they support heterosis exploitation, or they can reduce the diversity in gene complexity build-up in elite inbred germplasm are not to be preserved.

## Limitations in MAS

i. Marker-assisted selection methods are more costly. It needs a well-equipped laboratory viz expensive chemicals and equipment’s glassware.
ii. The detection of different linked DNA markers (such as RFLP, RAPD, AFLP, SNP, SRP, etc.), is more time consuming, difficult, and much laborious.
iii. Molecular marker-assisted selection requires the trained manpower in handle the sophisticated equipment, DNA isolation, and study of DNA markers
iv. The utilization of MAS is very difficult in QTL study due to their cumulative effects, which are greatly affected by environmental factors and genetic background.
v. Sometimes, marker-assisted selection (MAS) uses radioisotopes for labelling the DNA, which may lead to serious hazards for health. This is a major disadvantage of markers based on RFLP. However, markers based on PCR are safe in this regard.
vi. It has been reported from this study that marker-assisted selection may become least efficient than the selection of phenotypes in the long term.

## Conclusions

The most recent 30 years have seen a continuous improvement in the molecular marker technique from restriction fragment length polymorphism (RFLP) to single nucleotide polymorphism (SNPs) and the diversity of array technology based on molecular markers. Headways in the sequencing advancements have prompted the improvement of NGS stages that are minimal effort with high throughput. The fundamental purpose for this lies in erroneous phenotyping. Several efforts have been constructed to generate precise, new, and more efficient markers in plant breeding for agricultural importance (e.g. rice, maize, potato), but less research has been performed for developing markers in underutilized crops. Other scientific fields such as phylogenetics and molecular ecology have little information about the molecular marker techniques still now. The CRISPR technology has changed plants’ reproducing and hereditary qualities, and analysts are concentrating on altering the genomes of all financially significant plants. The major disadvantage of some methods in MAS is the need for preliminary information of the genome, in some cases, which requires additional and excess time-consuming laboratory work. It can be anticipated that most of the methods discussed in this review article could provide a structured database which could be utilized alone or in a mix with sequence-level characters in specific fields of plant science where they have not yet been used.

## Data Availability

Not applicable

## Abbreviations

AFLP: Amplified fragment length polymorphism
CAPS: Cleavage-amplified polymorphic sequence
CRISPR: Cluster regularly interspaced short palindromic repeat
DALP: Direct amplification of length polymorphism
iPBS: Inter primer binding site
ISAP: Inter SINE amplified polymorphism
iSNAP: Inter small RNA polymorphism
MAS: Marker-assisted selection
PAAP: Promoter anchored amplified polymorphism
QTL: Quantitative trait loci
RAD: Restriction site-associated DNA sequencing
RAPD: Random amplified polymorphic DNA
RBIP: Retrotransposon-based insertion polymorphism
REMAP: Retrotransposon microsatellite amplified polymorphism
RFLP: Restriction fragment length polymorphism
RGAP: Resistance gene analogue polymorphism
SCoT: Start codon targeted
SNP: Single nucleotide polymorphism
SSR: Simple sequence repeat
TFM: Targeted fingerprinting markers
TILLING: Targeted induced local lesions in genome
TRAP: Targeted region amplified polymorphism
